# Wounds, Antimicrobial Resistance and Challenges of Implementing a Surveillance System in Myanmar: A Mixed-Methods Study

**DOI:** 10.3390/tropicalmed6020080

**Published:** 2021-05-18

**Authors:** Win-Pa Sandar, Saw Saw, Ajay M. V. Kumar, Bienvenu Salim Camara, Myint-Myint Sein

**Affiliations:** 1 Department of Health Behaviour and Communication, University of Public Health, Yangon 11131, Myanmar; 2 Department of Medical Research, Ministry of Health and Sports, Yangon 05081, Myanmar; awsaw@mohs.gov.mm; 3 International Union Against Tuberculosis and Lung Disease, South-East Asia Office, New Delhi 110016, India; akumar@theunion.org; 4 International Union Against Tuberculosis and Lung Disease, 75006 Paris, France; 5 Yenepoya Medical College, Yenepoya (Deemed to be University), Mangaluru 575018, India; 6 Centre National de Formation et de Recherche en Santé Rurale de Maferinyah, Forécariah 4090, Guinea; bscamara@maferinyah.org; 7 Department of Microbiology, University of Medicine, Magway 04011, Myanmar; mmseindr@gmail.com

**Keywords:** SORT IT, operational research, GLASS, antimicrobials, wound infection, AMR surveillance

## Abstract

Wound infections with drug-resistant bacteria lead to higher mortality and morbidity and increased healthcare costs. We aimed to describe the spectrum of bacterial pathogens, isolated from wound cultures in Yangon General Hospital in 2018, and their antimicrobial resistance (AMR) patterns and to understand the challenges in implementing an AMR surveillance system in Myanmar. We conducted a concurrent mixed-methods study involving analysis of surveillance data and in-depth interviews with nine key personnel involved in AMR surveillance. Of 1418 wound specimens processed, 822 (58%) were culture-positive. The most common Gram-positive bacteria were coagulase-negative staphylococci (23.3%) and *Staphylococcus aureus* (15.1%). Among Gram-negative bacteria, *Escherichia coli* (12.5%) and *Pseudomonas aeruginosa* (10.1%) were common. *Staphylococcus aureus* isolates were resistant to penicillin (98%), oxacillin (70%) and tetracycline (66%). *Escherichia coli* showed resistance to ampicillin (98%). Lack of dedicated and trained staff (microbiologist, technician, data entry operator), lack of computers at sentinel sites and non-uniform and non-standardized data capture formats were the major challenges in implementing AMR surveillance. These challenges need to be addressed urgently. We also recommend periodic analysis and sharing of antibiograms at every hospital to inform the treatment regimens used in wound management.

## 1. Introduction

Antimicrobial resistance (AMR) has become a growing threat to human life globally, especially in developing countries [1]. The increasing incidence of AMR has been attributed to patients’ behavior of not completing a course of treatment or non-adherence to their treatment schedule; the challenges of supply chain management of antimicrobials in the population, leading to interruptions in treatment; unregulated use of antimicrobials, including their availability over-the-counter, and, most importantly, the irrational prescribing practices of healthcare professionals [2]. In the United States, more than 2.8 million antibiotic-resistant infections occur each year, and more than 35,000 people die as a result [3]. The data on AMR from low- and middle-income countries (LMICs) are still limited. The O’Neill Report estimated that deaths due to AMR could rise from approximately 700,000 deaths a year to close to 10 million deaths per year by 2050 [4].

One of the most frequent indications for antimicrobial treatment is wound infection, which is very common in settings with poor infection prevention and control (IPC) measures [5,6]. The burden of antibiotic-resistant infections across different wound types and care settings has been increasing in these settings [7]. The predominant bacterial isolates from the infected wounds include *Staphylococcus aureus, Escherichia coli, Proteus* species, *Klebsiella pneumoniae, Pseudomonas aeruginosa* and *Acinetobacter baumannii* [8,9,10,11].

Wound infections with antibiotic-resistant bacteria may lead to higher mortality and prolonged debility of the patient, which may result in a longer hospital stay and increased healthcare costs. The situation is significantly worse in developing countries due to irrational use of antibiotics [12]. In addition, in settings with poor IPC measures, inappropriate management of wound infections might contribute to the spread of pathogens to other patients and health providers. Therefore, improving in-hospital management of wound infections is key to prevent both the development and in-hospital transmission of resistant pathogens [13].

Better management of wound infection requires accurate diagnosis of infection and its resistance patterns, drug-sensitivity-guided treatment, host factors that influence antimicrobial activity and better management of adverse drug effects [14]. All these pre-conditions can be met only with a good antimicrobial surveillance system. In 2015, the World Health Organization (WHO) launched the Global AMR Surveillance System (GLASS) to support a standardized approach to the collection, analysis and sharing of data on AMR at a global level, in order to inform decision-making, drive local, national and regional action and provide the evidence base for action and advocacy [15]. However, there have been challenges in implementing an antimicrobial surveillance system in developing countries, where laboratory capacities are poor and surveillance networks in the health systems are weak or absent [16].

Myanmar joined the GLASS in 2018, and surveillance data have been captured since 2019. Anecdotal evidence indicates several challenges in implementing the AMR surveillance system. A PubMed search revealed no study from Myanmar exploring challenges of implementing an AMR surveillance system, with a focus on wound infection management. Hence, we conducted this study to describe the spectrum of bacterial pathogens isolated from positive wound cultures and their antimicrobial drug sensitivity patterns at one sentinel site and to understand the challenges in implementing the AMR surveillance system in the entire country and possible solutions to address them from the perspective of healthcare providers.

## 2. Materials and Methods

### 2.1. Study Design

This was a concurrent mixed-methods study with a quantitative (cross-sectional study involving secondary analysis of surveillance data) and qualitative component (descriptive study involving key informant interviews).

### 2.2. Setting

#### 2.2.1. General Setting

The Republic of the Union of Myanmar, located in South-East Asia, has a population of 51.4 million and is divided administratively, into Nay Pyi Taw Council territory, seven States and seven Regions.

Healthcare services are mainly funded by the government. As of 2014−2015, government health expenditures were estimated to account for 0.99% of the gross domestic product and 3.38% of general expenditures.

In Myanmar, public healthcare services are provided at three levels: primary, secondary and tertiary health facilities. The primary-level health facilities include sub-township and station hospitals (16 to 25 beds), rural health centers and sub-rural health centers. The secondary level includes regional or state hospitals, district hospitals (200 to 500 beds) and township hospitals (25 to 100 beds). The tertiary level consists of four general hospitals (up to 2000 beds), 50 specialist hospitals and teaching hospitals (100 to 1200 beds).

#### 2.2.2. AMR Surveillance in Myanmar

The National Action Plan (NAP) to combat AMR was developed in 2017 by adopting the five strategic objectives (awareness generation, surveillance, IPC, antimicrobial usage and research and innovation) of the global AMR guidelines. The National Multi-Sectorial Steering Committee (NMSC) is the national nodal agency for AMR in Myanmar. There are five technical working groups (TWGs) under NMSC to implement the five strategic objectives. The National Health Laboratory acts as a national coordinating center (NCC) to coordinate between NMSC and five TWGs for combating AMR. Surveillance for AMR is being carried out at seven sentinel laboratories, which include the National Health Laboratory, Yangon General Hospital, New Yangon General Hospital, North Okkalapa General Hospital, Thingangyan General Hospital, Mandalay General Hospital and the Nay Pyi Taw 1000-bed Hospital, in line with WHO GLASS. In these sentinel sites, routine culture and drug susceptibility testing is performed on various types of specimens. The data are recorded in both paper-based registers and a WHO electronic database (WHONET). The AMR data from all sentinel sites are sent to NCC bi-annually. NCC compiles all the data, validates them and then uploads them to the GLASS annually.

#### 2.2.3. Specific Setting

The present study was conducted at Yangon General Hospital (YGH), a 2000-bed government tertiary care teaching hospital with more than 24 specialties which provides treatment to patients from all over the country. This is one of the seven sentinel sites for AMR surveillance. As of 2018, an average of 3000 patients sought treatment at this hospital on a daily basis. In the same year, the wound cultures were positive in around 1000 patients from different wards, including medical wards, surgical wards, the orthopedic ward and the oncology ward.

#### 2.2.4. Procedure for Wound Cultures

In general, swabs are collected from the wounds suspected to be infected and are sent for culture and sensitivity to the Microbiology Department. Once received, the specimens are smeared on clean glass slides, stained with Gram stain and examined for morphology under microscopy. Then, they are cultured on MacConkey agar and blood agar media under aseptic conditions and incubated aerobically at 37 °C overnight. Visual inspection of the bacterial colonies indicates culture positivity. The suspected organism is then identified by Gram’s stain and a series of biochemical tests, including a catalase test, Mannitol Salt Agar with Egg Yolk and Vitek 2 GP (VITEK 2 automated ID/AST machine). Next, the strains are identified using procedures recommended by the Clinical and Laboratory Standards Institute (CLSI). At the last stage, the antibiotic susceptibility pattern is detected using Vitek 2 AST GP 67 (bioMérieux, Singapore). The antimicrobial drug susceptibility tests are performed depending on the organism isolated.

#### 2.2.5. Definitions

*Wound infection*: Wound infection is the deposition and multiplication of bacteria in tissue with an associated host reaction [17].

Positive wound culture: A positive culture means that bacteria grew after inoculating on the MacConkey agar and blood and under aseptic conditions at 37 °C for 24 h.

*Antimicrobial resistance (AMR):* AMR is the ability of a microorganism (such as bacteria, viruses and some parasites) to stop an antimicrobial (such as antibiotics, antivirals and antimalarials) from working against it [18].

### 2.3. Study Population

**Quantitative part:** We included all patients with suspected wound infections, whose wound swabs were processed for culture and drug sensitivity at the YGH from 1st January, 2018 to 31st December, 2018.

**Qualitative part:** Healthcare providers who are involved in the implementation of AMR surveillance and are responsible for all the sentinel sites in the country were interviewed. We used purposive sampling to select the interviewees so that providers involved at the different levels of the surveillance system were represented. They included one senior consultant microbiologist and one laboratory technician from the Microbiology Department of YGH, one first assistant from the surgical unit of YGH, one consultant microbiologist from the National Health Laboratory (NHL) and one person from each of the five technical working groups of the global AMR guidelines. The interviews were conducted from December 2019 to January 2020.

### 2.4. Data Variables, Sources of Data and Data Collection

**Quantitative data:** Data on aggregate number of wound cultures processed during the year 2018 were extracted from the paper-based register maintained by the Microbiology Department of the Yangon General Hospital, Myanmar. The aggregate numbers of positive cultures were extracted from the WHONET electronic database. Other variables included age, sex, bacterial isolates by Gram stain and antibiotic resistance pattern (resistant, intermediate, susceptible) for each drug tested. Data collection was done by the principal investigator with the help of a trained research assistant.

**Qualitative data:** Nine key informant interviews were conducted in the local language (Burmese) using an interview guide (Appendix A). The interview guide included open-ended questions to explore challenges in implementing the AMR surveillance system and suggestions to improve it. After obtaining informed consent, the interviews were audio-recorded. For two participants, who did not consent to audio recording, notes were taken by trained note takers. All the interviews were conducted by the principal investigator (a female medical doctor trained and experienced in qualitative research methods). While the interviewer was knowledgeable about the AMR surveillance system, she was not involved in its implementation—this enabled objectivity in data collection and analysis. The average duration of interviews was 60 min.

### 2.5. Analysis and Statistics

**Quantitative data:** We extracted the data from WHONET in Microsoft Excel^®^ format, checked for any inconsistencies and cleaned for analysis. We summarized the data using frequencies and percentages (for categorical variables) or means and standard deviations (for continuous variables). Resistance patterns of bacterial isolates were presented as bar charts.

**Qualitative data:** A manual thematic analysis was performed. All the audio recordings were transcribed verbatim and familiarization of the data was attained by repeated reading. Then, codes were developed by generating both inductive and deductive codes until no new code emerged. After codes were developed, themes were identified in consultation with two co-authors who also had access to all transcripts. Qualitative data analysis was initially conducted in Burmese and the final results were translated into English. The findings were reported according to “Consolidated Criteria for Reporting Qualitative Research” (COREQ) guidelines [19].

## 3. Results

### 3.1. Quantitative Findings

Overall, 1418 wound cultures were processed during the study period. Of them, 822 (58%) were culture-positive. The age of the patients ranged from 3 months to 96 years, with the mean (SD) age of 46.4 (18.4) years, and 462 (56%) were males (Appendix B).

Among the positive cultures, 696 (85%) grew mono-microbial isolates, and 126 (15%) had poly-microbial growth. A total of 1014 isolates were obtained, of which 951 (94%) were pathogens and 63 (6%) were skin commensals. Out of 951 pathogens, 442 (43.6%) were Gram-positive organisms, and 509 (50.2%) were Gram-negative organisms. The most commonly isolated Gram-positive organisms were coagulase-negative staphylococci (236, 23.3%) followed by *Staphylococcus aureus* (153, 15.1%). Among Gram-negative organisms, *Escherichia coli* and *Pseudomonas aeruginosa* were observed in 127 (12.5%) and 102 (10.1%) isolates, respectively (Table 1).

**Figure 1:**
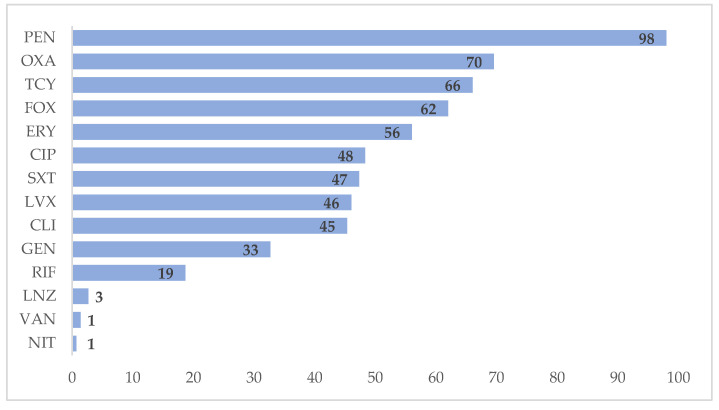
Antimicrobial resistance patterns in *Staphylococcus aureus* isolates at Yangon General Hospital, Myanmar, in 2018. X-axis represents the percentage of resistance. CIP = Ciprofloxacin, CLI = Clindamycin, ERY = Erythromycin, FOX = Cefoxitin, GEN = Gentamicin, LNZ = Linezolid, NIT = Nitrofurantoin, OXA = Oxacillin, PEN = Penicillin, RIF = Rifampin, SXT = Trimethoprim/Sulfamethoxazole, TCY = Tetracycline, VAN = Vancomycin.

**Figure 2:**
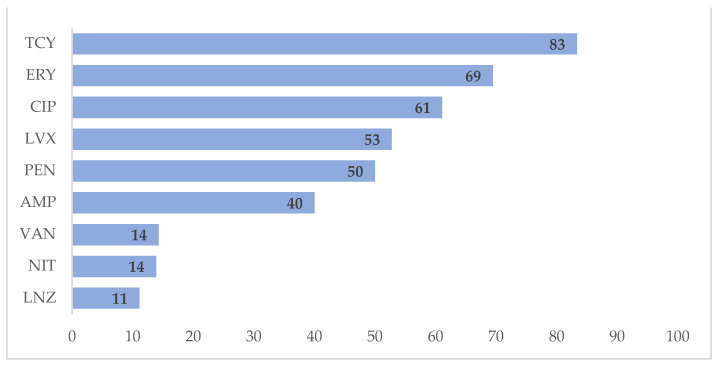
Antimicrobial resistance patterns in *Enterococcus* spp. isolates at Yangon General Hospital, Myanmar, in 2018. X-axis represents the percentage of resistance. AMP = Ampicillin, CIP = Ciprofloxacin, ERY = Erythromycin, GEN = Gentamicin, LNZ = Linezolid, NIT = Nitrofurantoin, PEN = Penicillin, TCY = Tetracycline, VAN = Vancomycin.

Among isolates of *Staphylococcus aureus,* 98% were resistant to penicillin, 70% were resistant to oxacillin and 66% were resistant to tetracycline (Figure 1). Among *Enterococcus* spp. isolates, 83% were resistant to tetracycline (83%), followed by erythromycin (69%) and ciprofloxacin (61%) (Figure 2). We have not reported the antibiotic resistance pattern for coagulase-negative staphylococci and *Streptococcus* spp. because of incompleteness of data.

**Figure 3:**
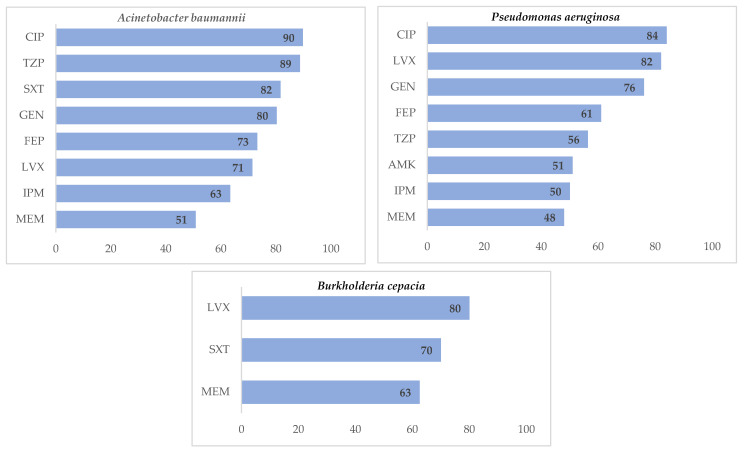
Antimicrobial resistance patterns in non-Enterobacteriaceae isolates at Yangon General Hospital, Myanmar, in 2018. X-axis represents the percentage of resistance. AMK = Amikacin, CIP = Ciprofloxacin, FEP = Cefepime, GEN = Gentamicin, IPM = Imipenem, LVX = Levofloxacin, MEM = Meropenem, SXT = Trimethoprim/Sulfamethoxazole, TZP = Piperacillin/Tazobactam.

Of all *Acinetobacter baumannii* isolates, 90% were resistant to ciprofloxacin, 89% to piperacillin/tazobactam and 82% to trimethoprim/sulfamethoxazole. *Pseudomonas aeruginosa* showed resistance to ciprofloxacin (84%), levofloxacin (82%) and gentamicin (76%). *Burkholderia cepacia* was commonly resistant to levofloxacin (80%), trimethoprim/sulfamethoxazole (70%), and Meropenem (63%) (Figure 3).

**Figure 4:**
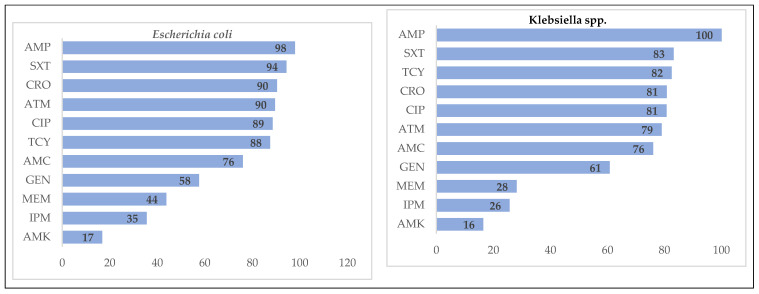

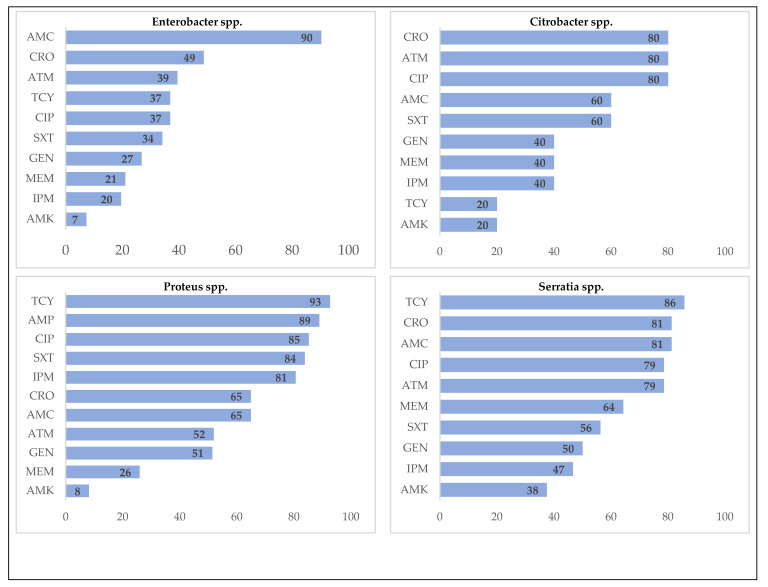
Antimicrobial resistance patterns in Enterobacteriaceae isolates at Yangon General Hospital, Myanmar, in 2018. X-axis represents the percentage of resistance. AMK = Amikacin, AMC = Amoxicillin/Clavulanic acid, AMP = Ampicillin, ATM = Aztreonam, CIP = Ciprofloxacin, CRO = Ceftriaxone, GEN = Gentamicin, IPM = Imipenem, MEM = Meropenem, SXT = Trimethoprim/Sulfamethoxazole, TCY = Tetracycline.

*Escherichia coli* were resistant most commonly to ampicillin (98%), trimehoprim/sulfamethoxazole (94%) and ceftriaxone (90%). *Klebsiella* spp. showed 100% resistance to ampicillin; they were also commonly resistant to trimethoprim/sulfamethoxazole (83%), ceftriaxone (82%) and aztreonam (81%). Among *Enterobacter* spp., 90% showed resistance to amoxicillin/clavulanic acid. Eighty percent of *Citrobacter* spp. isolates were resistant to ceftriaxone, aztreonam and ciprofloxacin. Resistance to tetracycline, ampicillin and ciprofloxacin was observed in 93%, 89% and 85% of *Proteus* spp. Isolates, respectively. Among *Serratia* spp., tetracycline (86%), ceftriaxone (81%) and amoxicillin/clavulanic acid (81%) were the most commonly resistant antibiotics (Figure 4).

### 3.2. Qualitative Findings

A total of nine participants from various departments (three from Yangon General Hospital, one from NHL and five members of the NMSC) were interviewed. They represented a wide range of designations (from laboratory officer to Professor and Director of respective departments) and work experience (1−25 years). The participants’ age ranged from 36 to 59 years, and all but one were female.

#### 3.2.1. Challenges in Implementing the AMR Surveillance System

The majority of the participants expressed that limited resources were the main reason for the lack of good-quality data on AMR. One challenge was related to a lack of human resources (insufficient number of microbiologists, pharmacists, medical technicians, data entry staff). Some reported that there was no regular focal person for AMR at sentinel sites. Lack of computers was cited as another challenge.

Most of the participants pointed out that there was no uniformity in the laboratory forms used for the collection of specimens, and these forms were incomplete and lacked background information on specimens, which made it a challenge when entering data into the WHONET software. There were challenges in compiling surveillance data from seven sentinel sites because of the non-uniform and non-standardized data capture formats.

“*We have not standardized the configuration (laboratory configuration for antimicrobials test and data entry) and we only taught them how to build configuration. Each hospital built their configuration as they liked and therefore, they are not uniform with each other. Therefore, when we hold the meeting this time, we will discuss how to standardize the configuration style and the antibiotic list*.”

(Participant 6)

A few participants (Participants 8 and 9) stated that their workload was heavy due to limited manpower and hence they were unfamiliar with AMR data.

“*My department’s workload is heavy, and only one microbiologist, one officer and one medical technician were present…… Ask my officer if you want to know more about data. I don’t know*.”

(Participant 9)

According to some participants, there was no nationwide research on AMR. Some mentioned that there were plans to conduct national studies following the pilot phase, but these were not realized due to lack of funding. Some participants also highlighted the challenge of non-availability of secondary data in electronic format for researchers to use.

“*For the reviews and research, the data are not readily available. To know how many organisms there are, I must count from the registration book and make a tally. Electronic data does not exist. That makes me have a headache*.”

(Participant 5)

#### 3.2.2. Suggestions to Improve AMR Surveillance 

The participants provided many suggestions to improve the AMR surveillance system—these included strengthening its capacity and establishing a proper data management and reporting system.

##### Strengthening Capacity for Surveillance

All participants stated that there was a need for developing the capacity of staff for various activities. One participant emphasized that a well-trained microbiologist should be assigned at every sentinel site. Most participants suggested that dedicated staff for data entry and data management be recruited, trained and retained.

“*It is required to get software training for management and analysis of AMR data. Microbiologists also need capacity building for AMR surveillance…. Major constraint is technical*.”

(Participant 4)

“*This hospital uses computerized ICD code 10 for diagnosis reporting system. Like that, if we have a proper software for the collection of patient’s data, it would be very convenient for us. Or we should have one data collector for this*.”

(Participant 7)

##### Use of Hospital Antibiogram Data 

Some participants described the importance of antibiograms and suggested that there should be an antibiogram for each and every hospital, prepared and shared periodically, to identify emerging resistance patterns and adjust the use of antibiotics accordingly. They also noted that clinical settings are known for high antimicrobial use and, in turn, infection prevention and control, especially in the context of hospitals, was an important aspect in controlling AMR.

“*We publish antibiogram data biannually or annually which are already sent to NHL by our microbiologist. We send these data to every ward in the hospital so that they can take actions. We can adjust the antibiotic usage by reviewing this antibiogram data…So, every hospital should have antibiogram*.”

(Participant 3)

##### Standardized Guidelines and Formats 

Most participants mentioned that there should be a national body for AMR surveillance and a national antibiotic guideline. They also suggested use of standardized and uniform formats for data collection and capture.

“*It would be nice if something like a proforma can be distributed evenly. In that form, all the necessary information like background information, and diagnosis should be included. The format needs to be consistent*.”

(Participant 3)

##### Multisectoral Approach

A few participants stated that antimicrobials were used not only for human beings but also for plants and animals and, hence, strong collaboration with other departments and ministries, such as the Ministry of Health and Sports (MoHS), the Food and Drug Administration (FDA) and the livestock and agriculture sectors, was mandatory for a One Health approach. They also stated that legislation to ban over-the-counter sales and purchases of drugs should be enforced.

“*Antibiotics are widely used not only in human sector but also in animal, fishery and agricultural sectors. We need to carefully think about how to do legislation on antibiotic use in collaboration with FDA. There should be strong multisectoral collaboration to control irrational use of antibiotics like taking antibiotics without prescription and not complete the full course of antibiotics*.”

(Participant 4)

## 4. Discussion

The present study, combining quantitative and qualitative methods, is the first of its kind to examine antimicrobial resistance in wound infections in Myanmar. It described bacterial isolates from positive wound cultures, and their antimicrobial resistance patterns. This knowledge will aid healthcare providers in choosing the appropriate antibiotics for the management of wound infections in Myanmar and countries with similar contexts.

In this study, over half (58%) of the analyzed wound cultures showed bacterial growth. This result was found to be consistent with a study conducted in India, which showed that 59.8% of the wound cultures had bacterial growth [20]. However, other studies conducted in Nigeria and Tanzania reported that bacterial growth was observed in the majority of wound swabs (91.4% and 83.1%) [21,22]. These differences in culture positivity might be due to the differences in the hospital settings and implementation of infection prevention and control measures.

We found mono-microbial isolates in the majority of the positive cultures, and only 15% showed poly-microbial isolates in this study. In studies conducted in India and Ethiopia, similar results were found [8,10,20]. According to the present study, Gram-positive and Gram-negative organisms were 43.6% and 50.2%, respectively. This result was also similar to the studies performed in India and Ethiopia [10,20].

According to the present study, the most common bacterial isolates were CoNS, *Staphylococcus aureus* and *Escherichia coli*. In other studies, the most common bacteria were *Staphylococcus aureus* [8,10,22,23]. Identifying common bacteria in wound infections can allow for the determination of causative isolates for healthcare providers in remote or resource-limited settings.

According to the antimicrobial resistance pattern of this study, nearly all *Staphylococcus aureus* isolates were resistant to penicillin, and 70% of isolates were resistant to oxacillin. A study conducted in Tanzania showed that resistance to amoxicillin was observed in 61.9% of cases [22]. The 2019 WHO AWaRe classification of antibiotics indicates that penicillin has activity against a wide range of commonly encountered susceptible pathogens. This study found an increased rate of resistance to these groups. This might be due to over-the-counter availability, misuse and overuse of these antibiotics among general practitioners and community members prior to hospital admission. *Escherichia coli* was most resistant to ampicillin, which is in line with findings from India and Ethiopia [10,20].

This is the first study to explore challenges in implementing an AMR surveillance system in Myanmar. Key informants involved in AMR surveillance reported AMR surveillance-related challenges. The most common challenges were a lack of capacity and a lack of infrastructure and human resources in hospitals and sentinel sites. A study on AMR surveillance in low- and middle-income countries also pointed out the weakness in laboratory capacity and infrastructure in the AMR surveillance system, especially in the public sector [24]. If we do not have well-equipped laboratories at the hospitals and sentinel sites, we cannot create a well-functioning surveillance system for AMR. Thus, we need to address these issues urgently.

Another challenge reported by this study was lack of a regular, well-trained microbiologists at the sentinel sites, because of frequent turnover. This is in line with the findings of a study from Ethiopia, which reported frequent turnover of microbiology staff at the surveillance sites, leading to challenges in electronic data entry [25].

There are a number of implications for policy and practice. First, based on the quantitative findings, we identified the most common bacterial isolates in wound infections and their resistance patterns in YGH. This will help doctors in choosing the best empiric antibiotic therapy. The qualitative results also highlighted that every hospital should have an antibiogram, so we recommend that antibiograms be published and updated periodically in order to support the healthcare team in selecting the suitable antibiotics for patients. This will contribute to the implementation of a national antibiotic policy and guidelines.

Second, we found that the bacteria were most resistant to the access group antibiotics, reflecting the irrational use of antibiotics. Therefore, the importance of antimicrobial stewardship and rational antimicrobial use must be included in the medical school curricula in order to improve the prescribing practices of physicians and control antimicrobial resistance.

Third, the interviews found that there were inconsistencies in data capture formats. While this is not particularly a limitation when reviewing the data from a single site, this becomes an important limitation while trying to compile the data at the national level. The formats need to be standardized as soon as possible and all the concerned staff should be trained in using these formats. This will require commitments from the responsible persons from MoHS, including Senior Medical Superintendents (SMSs) and people in charge of the different wards of hospitals.

Fourth, dedicated and trained human resources (especially microbiologists and data entry operators) must be made available at all sentinel sites. Computers need to be provided with all the software installed so that there are no challenges in data entry and transmission of reports to national level.

Fifth, the present study was focused on wound data from one sentinel site. Future studies should consider studying AMR at all sentinel sites in the country. This will help in obtaining nationally representative information about AMR, which may be of help in establishing a national antibiotics policy and guidelines.

There were several strengths in this study. First, we used a mixed-methods study design. The quantitative component investigated the antimicrobial resistance pattern among wound infections, and the qualitative component helped to elucidate the challenges faced in implementing an AMR surveillance system. Second, we used the routinely collected data and hence the findings reflect the ground realities. Finally, we adhered to the Strengthening The Reporting of Observational Studies in Epidemiology (STROBE) guidelines for reporting the quantitative component and COREQ guidelines for the qualitative component of the study.

The study also had a few limitations, which were related to incompleteness and inconsistencies of routinely captured data in WHONET. These could not be checked and corrected.

## 5. Conclusions

In conclusion, we found that nearly six in ten wound specimens procured at Yangon General Hospital in Myanmar in 2018 were culture-positive. The most common bacteria identified were coagulase-negative staphylococci, *Staphylococcus aureus, Escherichia coli* and *Pseudomonas aeruginosa*. The resistance patterns of these bacteria were described. The lack of dedicated and trained staff (microbiologists, technicians, data entry operators) and computers at sentinel sites and the non-uniform and non-standardized data capture formats were the major challenges in implementing AMR surveillance. These challenges need to be addressed urgently. We also recommend periodic analysis and sharing of antibiograms at every hospital to inform the treatment regimens used in wound management.

## Figures and Tables

**Table 1:** Bacterial isolates from wound cultures at Yangon General Hospital, Myanmar, in 2018.

Organism Groups	Organisms	Number	(%)
**Total (isolates)**	1014 *	**100**
**Gram-positive**	*Staphylococcus aureus*	153	15.1
	Coagulase-negative staphylococci	236	23.3
	*Streptococcus* spp.	16	1.6
	*Enterococcus* spp.	37	3.6
**Gram-negative**	**Enterobacteriaceae**		
	*Escherichia coli*	127	12.5
	*Klebsiella* spp.	80	7.9
	*Enterobacter* spp.	43	4.2
	*Citrobacter* spp.	6	0.6
	*Proteus* spp.	37	3.6
	*Serratia* spp.	16	1.6
	**Non-Enterobacteriaceae**		
	*Acinetobacter baumannii*	72	7.1
	Other *Acinetobacter* spp.	5	0.5
	*Pseudomonas aeruginosa*	102	10.1
	Other *Pseudomonas* spp.	11	1.1
	*Burkholderia cepacia*	10	1.0

* Only 951 included here as 63 are skin commensals.

**Figure A1:**
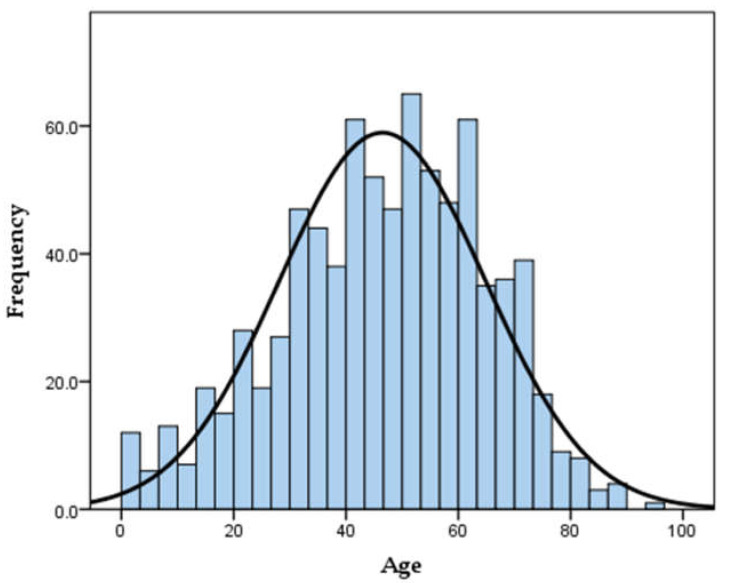
Age distribution of study participants at Yangon General Hospital, Myanmar, in 2018.

**Figure A2:**
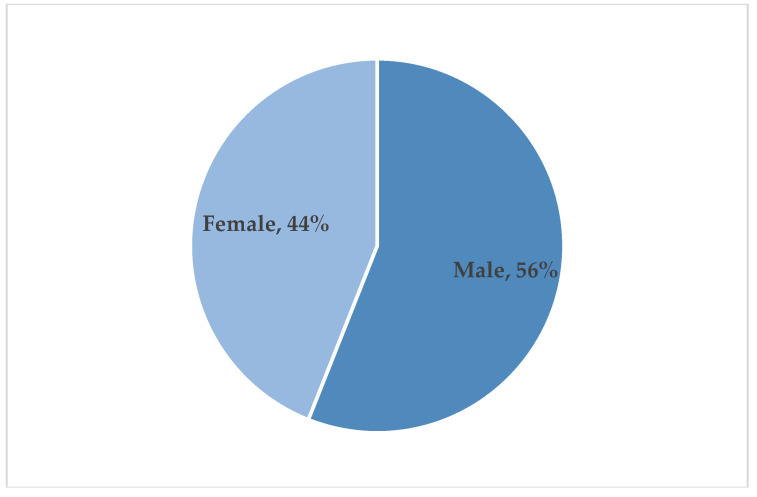
Sex distribution of study participants at Yangon General Hospital, Myanmar, in 2018.

## Data Availability

The data that support the findings of this study are available from the corresponding author, W.P., upon reasonable request.

## Open Access Statement

In accordance with WHO’s open-access publication policy for all work funded by WHO or authored/co-authored by WHO staff members, the WHO retains the copyright of this publication through a Creative Commons Attribution IGO license (http://creativecommons.org/licenses/by/3.0/igo/legalcode) which permits unrestricted use, distribution and reproduction in any medium provided the original work is properly cited. In any use of this article, there should be no suggestion that WHO endorses any specific organization, products or services. The views expressed in this article are those of the authors and do not necessarily reflect those of their affiliated institutions. The use of the WHO logo is not permitted. This notice should be preserved along with the article’s original URL.

## Appendix A

### Appendix A.1. Key Informant Interview: Interview Guide

Name of the participant:

Designation:

Date of Interview:

Interview start/end time:

Name of the Interviewer:

Notetaker:

### Appendix A.2. Introduction

Introduce yourself and the notetaker and explain who we are and what we’re trying to do.

We are conducting research on “Surveillance of antimicrobial resistance in wound infections at Yangon General Hospital, Myanmar in 2018: A mixed-methods study”.

We would like to ask some questions to help me understand challenges in implementing the AMR surveillance system and possible solutions to address them from the perspective of healthcare providers. We are very interested to hear your valuable opinion on that. There are no right or wrong answers to these questions.

The interview will take about 60 min. The information you give us is completely confidential and we will not associate your name with anything you say in the interview.

We would also like to ask your permission to **audio-record** our discussion. The recording will help me ensure that I don’t miss important things you say. It is difficult to record everything accurately using notes only. The recording will not include your name and will only be used by me. It will be deleted when the research is completed.

You may refuse to answer any questions or withdraw from the study at any time.

### Appendix A.3. General and Demographic Information

- *Please tell me about yourself*.
- Age, rank, level of education, role in the antimicrobial surveillance system, number of years in this system.

### Appendix A.4. Information Related to AMR Surveillance System

- Please describe your main routine activities, role, and responsibilities in the antimicrobial surveillance system
- Now let talk about the challenges you have been facing in carrying out properly these activities.
  o What challenges do you face regarding communication, data reporting, or data monitoring of the antimicrobial surveillance system?
  o What are the technical challenges faced in the antimicrobial surveillance system? As technical aspects, I refer to laboratory technical capacities, the competencies, availability, and motivation of human resources (physicians, nurses, laboratory technicians, and data reporting officers).
  o Could you also explain about the challenges related to the logistics aspects such difficulties in availability and management of technology for a functional surveillance system?
  o If there is any other challenge you or other staff have been facing regarding antimicrobial surveillance system, please explain.
- What measures have been put in place to address these challenges?

### Appendix A.5. Suggestions

- From your experience, what do you think are the possible feasible solutions to address these challenges?
- What concrete recommendations and to whom may you formulate to improve wound-related antimicrobial surveillance system in general?
- Is there any other information you would like to share with me? If yes please share.

### Appendix A.6. Conclusions

This is the end of our interview. I thank you so much for your time and the information provided.

In case I need further information from you, I will contact you again if you do not mind. In case you also need more information about the present study, please feel free to contact me at +95 9 5097201.
